# Solar Driven Photocatalytic Activity of Porphyrin Sensitized TiO_2_: Experimental and Computational Studies

**DOI:** 10.3390/molecules26113131

**Published:** 2021-05-24

**Authors:** Sebastian Otieno, Anabel E. Lanterna, John Mack, Solomon Derese, Edith K. Amuhaya, Tebello Nyokong, Juan C. Scaiano

**Affiliations:** 1 Centre for Advanced Materials Research (CAMaR), Department of Chemistry and Biomolecular Sciences, University of Ottawa, 10 Marie Curie, Ottawa, ON K1N 6N5, Canada; seba.otieno@gmail.com; 2 School of Pharmacy and Health Sciences, United States International University—Africa, Nairobi 00800, Kenya; 3 Department of Chemistry, University of Nairobi, Nairobi 00100, Kenya; sderese@uonbi.ac.ke; 4 School of Chemistry, University of Nottingham, University Park, Nottingham NG7 2RD, UK; Anabel.Lanterna1@nottingham.ac.uk; 5 Institute for Nanotechnology Innovation, Rhodes University, Makhanda 6139, South Africa; j.mack@ru.ac.za

**Keywords:** photocatalysis, porphyrin, electron injection, hydrogen generation, titanium dioxide

## Abstract

The absence of a secure long-term sustainable energy supply is recognized as a major worldwide technological challenge. The generation of H_2_ through photocatalysis is an environmentally friendly alternative that can help solve the energy problem. Thus, the development of semiconductor materials that can absorb solar light is an attractive approach. TiO_2_ has a wide bandgap that suffers from no activity in the visible spectrum, limiting its use of solar radiation. In this research, the semiconductor absorption profile was extended into the visible region of the solar spectrum by preparing porphyrin-TiO_2_ (**P**-TiO_2_) composites of *meso*-tetra(4-bromophenyl)porphyrin (**PP1**) and *meso*-tetra(5-bromo-2-thienyl)porphyrin (**PP2**) and their In(III), Zn(II) and Ga(III) metal complexes. Density functional theory (DFT) and time-dependent density functional theory (TD-DFT) calculations were performed on the porphyrins to gain insight into their electron injection capability. The results demonstrate that **P**-TiO_2_ systems merit further in-depth study for applications that require efficient photocatalytic H_2_ generation.

## 1. Introduction

Rapid growth in world population and increased industrial activities have led to a steady rise in global energy consumption [1]. According to the BP Statistical Review of World Energy, global energy consumption stands at 13.9 MTOE (Million Tonnes of Oil Equivalent). This figure is expected to nearly double by 2050 [1]. Currently, ~85% of global energy running the transport and industrial sector is supplied by fossil fuels. This has led to increased greenhouse gas (GHG) emissions and substantial depletion of the most easily exploited carbon-based energy resources that could alternatively have been used to produce valuable chemicals [2,3]. The key to reducing problematic emissions (NO_x_, SO_x,_ and GHGs) is to increase the efficiency of energy production and consumption while reducing the fossil fuel component in the energy mix [4,5]. A transition to hydrogen fuel is considered an ideal alternative to fossil fuels [4,5,6]. Hydrogen has potentially higher energy efficiency, high energy density and low generation of pollutants [7,8]. The world in the recent years has seen a steady transition from solid to liquid to gas fuels. Each successive source has increased the ratio of hydrogen to carbon. Hydrogen is an attractive chemical energy carrier that on reaction with oxygen, produces only water, thus a clean energy source.

Advanced oxidation processes (AOPs) such as photocatalytic hydrogen generation from sacrificial electron donors (SEDs) in aqueous media is one of the green sources being explored. This is due to the mild reaction conditions required, using only sunlight and a semiconductor [9,10]. Transition metal oxide systems such as TiO_2_ and ZnO have been the focus of appreciable research interest in hydrogen generation under UV-visible light excitation [11,12,13,14]. This application is based on the generation of an electron-hole (e-h) pair upon photoexcitation and the subsequent transfer of photoinduced electrons to electron acceptors such as protons through the metal oxide conduction band (CB) [10,15,16]. TiO_2_, in particular, has gained widespread applications in sunscreens, paints, photovoltaics and environmental remediation [17,18,19,20,21]. These applications are credited to its wide distribution in terms of its commercially exploitable reserves, inertness, favorable photocatalytic properties, low toxicity, and high photo efficiency and activity [22,23,24].

Despite many advantages, TiO_2_ is a wide bandgap semiconductor. Thus, it predominantly absorbs light in the UV region (<400 nm) of the solar spectrum, taking advantage of only 5% of the solar energy reaching the earth surface [22]. Low sensitivity to photons in the visible region, which constitutes about 41% of solar energy, poses a significant disadvantage to its use in solar panels [25]. On the other hand, rapid e-h recombination severely hinders practical efficiencies derived from any mechanism aimed at harvesting electrons or holes [18,19,20,21,22,23,24,25,26]. With these shortcomings in mind, researchers have focused on developing photoactive systems that will extend the photoresponse spectrum of TiO_2_ into the visible region and prolong the life span of charge-separated states [14,15]. Doping with narrow bandgap semiconductors, decorating TiO_2_ surface with nanoparticles, and dye sensitization are some of the modification strategies that have been used to shift the optical response of TiO_2_-based photocatalysts [18,22,27,28].

Recently, there has been a significant focus on dye sensitization, due to the difficulty in preparing doping materials and the need for lattice exchange at very high temperatures [22]. Upon photoexcitation of the dye, sufficient energy is produced that prompts surface electron transfer between the dye and TiO_2_ interface populating the conduction band (CB) [9,29]. Ruthenium-based sensitizers were extensively used initially. However, their practical applications have been limited due to their undesirable environmental impact leaving non-toxic organic dyes such as porphyrins as the most desirable alternative [30,31].

Here, we investigate the photocatalytic suitability of different porphyrins when adsorbed onto the TiO_2_ surface. This study aims to gain insights about the sensitization process using different porphyrin systems that could enhance the material’s efficiency. For this purpose, *meso*-tetra(4-bromophenyl)porphyrin (**PP1**) and *meso*-tetra(5-bromo-2-thienyl)porphyrin (**PP2**) and their In(III), Zn(II) and Ga(III) metal complexes were synthesized and characterized to examine whether modifying the *meso*-aryl groups has a significant impact on photocatalytic activity. The structural difference between *meso*-phenylporphyrins and *meso*-5-bromothien-2-ylporphyrins is known to result in significantly different photophysical and electrochemical properties [32]. Zinc, indium, and gallium metals were used to better understand trends in photocatalytic activity related to the central coordinated ion. Theoretical calculations were carried out using the density functional theory (DFT) and time-dependent density functional theory (TD-DFT) methods to develop a deeper understanding of the intrinsic optical properties of the porphyrins. The porphyrins were adsorbed on nanometric TiO_2_ to yield **P**-TiO_2_ nanocomposites. The photocatalytic activity of the **P**-TiO_2_ nanocomposites in hydrogen generation was investigated under irradiation by a solar simulator (AM 1.5). The results suggest that **P**-TiO_2_ photocatalysts merit further in-depth study for use in hydrogen generation applications.

## 2. Results and Discussion

### 2.1. Electronic Absorption Spectra of **PP1** and **PP2** and Their Metal Complexes

**Scheme 1:**
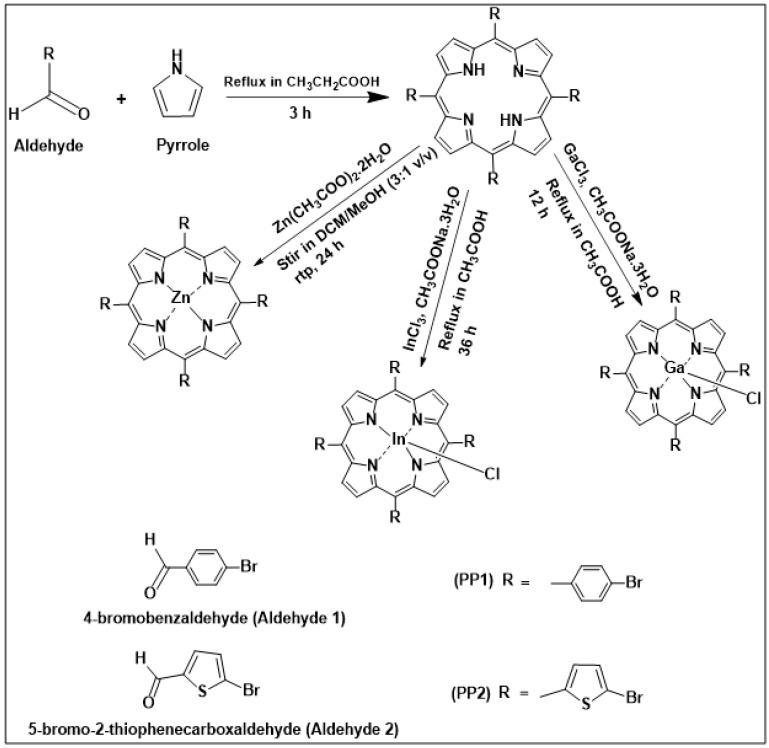
Synthetic pathway of **PP1**, **PP2** and their metal complexes.

The details for the synthesis of porphyrins are provided in the Supporting Information with ^1^H NMR and mass spectra provided in Figures S1–S4 and synthesis is shown in Scheme 1. The results suggest **PP1** and **PP2** are obtained as monomers and no evidence for dimerization/polymerization was detected.

**Figure 1:**
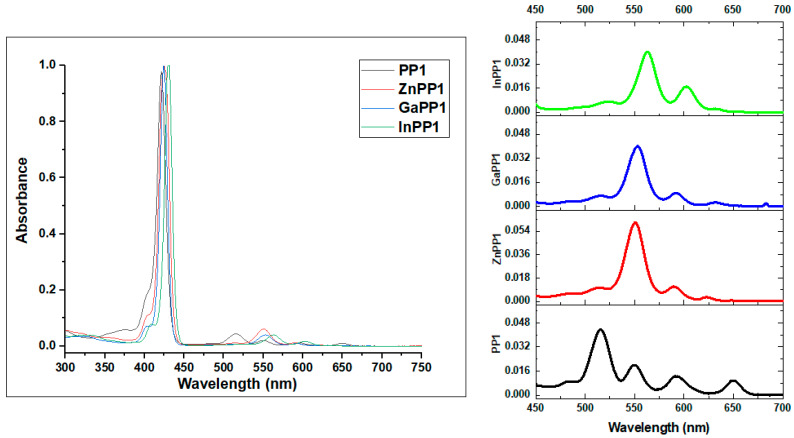
(**Left**) Normalized absorption spectra of **PP1** and the corresponding metal complexes in toluene. (**Right**) Spectra zoom in the Q band region.

**Figure 2:**
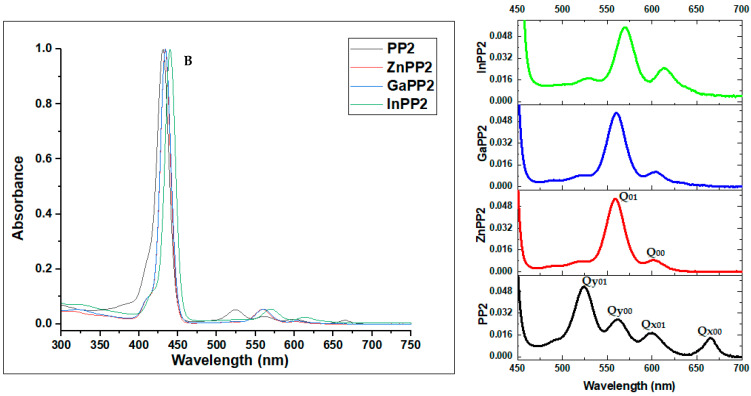
(**Left**) Normalized absorption spectra of **PP2** and the corresponding metal complexes in toluene. (**Right**) Spectra zoom in the Q band region.

UV-visible absorption spectra are one of the most informative spectroscopic methods in the context of porphyrin chemistry. **PP1**, **PP2** and their metal complexes all exhibit absorption spectra characteristic of *etio* type porphyrins with an intense Soret (or B) band that lies between 418−434 nm and weaker Q bands in the 500−700 nm region (Figure 1 and Figure 2).

**Figure 3:**
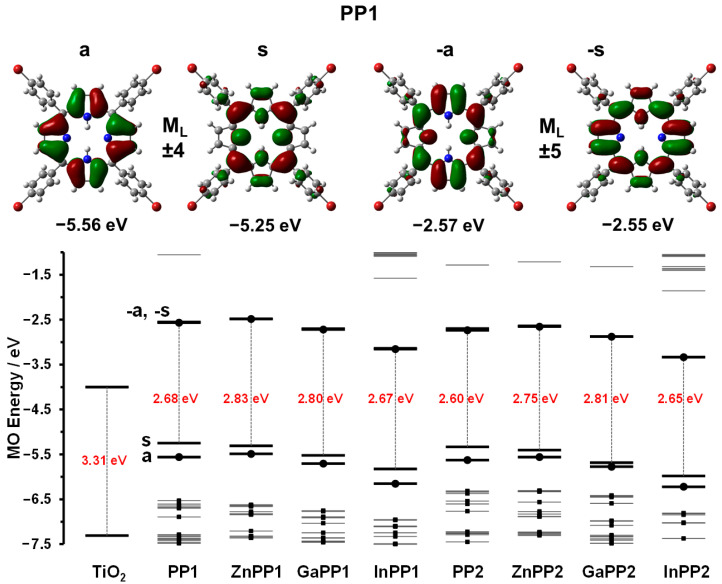
Angular nodal patterns and energies of the **a**, **s**, **−a** and **−s** MOs of **PP1** (TOP). Calculated MO energies and HOMO−LUMO gaps for TiO_2_ [31], **PP1**, **PP2** and the metal porphyrin complexes from the B3LYP geometry optimizations (BOTTOM). The **a**, **s**, **−a** and **−s** MOs are highlighted with thicker black lines, while other occupied MOs are denoted with a small black square. Black circles are used to highlight the **a** and **−a** MOs. The HOMO−LUMO gaps are denoted with vertical black dashed lines and the values are provided in red text.

Gouterman’s four-orbital model [33] can be used to rationalize trends in the electronic excitation spectra of free base porphyrins and their metal complexes. The main trends in the optical and redox properties can be rationalized through a consideration of four frontier orbitals that are derived from the highest occupied molecular orbital (HOMO) and lowest unoccupied molecular orbital (LUMO) of a C_16_H_16_^2−^ parent hydrocarbon perimeter. Michl introduced an **a**, **s**, **−a** and **−s** nomenclature (Figure 3) for these orbitals depending on whether there is a nodal plane (**a** and **-a**) or large coefficients (**s** and **-s**) aligned with the *y*-axis [34]. For example, the HOMO−1, HOMO, LUMO and LUMO+1 of **PP1** can be assigned as the **a**, **s**, **−a** and **−s** MOs, respectively (Figure 3). The MOs derived from the HOMO and LUMO of the C_16_H_16_^2−^ parent perimeter have M_L_ values of ±4 and ±5, respectively. Four spin allowed one-electron transitions are possible within the four-orbital model. It is on this basis that Gouterman predicted the possibility of allowed B transition (ΔM_L_ = ±1) at high energy and a forbidden Q transition (ΔM_L_ = ±9) at lower energy [33,35]. Free base porphyrins (**PP1** and **PP2**) have *D*_2h_ symmetry so there is a splitting of the main electronic Q_00_ transition and a Q_01_ overtone into *x*- and *y*-polarized components, so that four Q bands are observed [33,36]. However, metalloporphyrins have *D*_4h_ symmetry and hence have only two Q bands due to the main electronic transition and a vibrational overtone [33]. This reduction in the number of the Q bands is a characteristic feature of metalloporphyrins, further confirming the successful insertion of metal ions into the porphyrin core.

The experimental data provided in Table 1 demonstrate that there are only relatively minor shifts in the absorption bands of **PP1** relative to those of tetraphenylporphyrin (H_2_TPP), since the only structural difference is the bromine atom at the *para*-position of the phenyl rings. Phenyl moieties have only a minimal influence on the electronic structure of the porphyrins, due to the unfavorable steric interaction between β hydrogens of the pyrrole ring and *ortho* hydrogens of the phenyl group. This positions the phenyl substituent orthogonal to the porphyrin core, thus resulting in an increased rotational barrier [32,37].

However, *meso*-5-bromothien-2-yl-substituted **PP2** has spectral bands that are red shifted by 10 nm compared to the spectrum of **PP1** (Table 1). Smaller 5-membered ring *meso*-substituents such as the thienyl groups do not have *ortho* hydrogens, thus making it easier for the *meso*-aryl rings to adopt a coplanar orientation with the porphyrin core [38]. This enhances the resonance interactions with the tetrapyrrole ring, which results in a significant red shift of the Q and B bands of **PP2** [39,40]. This trend is also observed in the spectra of the metal complexes of **PP2** and **PP1** (Table 1**).**

The B bands for the metal derivatives of **PP1** and **PP2** are red shifted by 2–9 nm compared to those of their respective free base monomers (Table 1). Metal insertion can slightly alter the π electron delocalization of the porphyrin ring. Co-ordination of metals at the porphyrin core improves their structural symmetry since the inner NH protons tilt the pyrrole ring out of plane [34,38]. Therefore, greater resonance interaction with the *meso*-substituents is observed due to the reduced steric hindrance. The extended conjugation is reflected in bathochromic shift of the Q and B bands [37,41]. Among the metallated porphyrins, indium complexes exhibited the largest shifts of 8−9 nm, a scenario that has been reported previously [42]. Similar observations were observed in previous studies [41,43,44,45]. It is noteworthy that **InPP1** and **InPP2** were predicted to have the smallest HOMO−LUMO gap values in the geometry optimization calculations (Figure 3), which is consistent with the observed red shifts of the major spectral bands.

### 2.2. Photo-Physical Characterization of **PP1**, **PP2** and Their Metal Complexes

The synthesized porphyrins exhibit two characteristic porphyrin emission peaks that arise from the Q_00_ and Q_01_ transitions [35,46,47]. The emission spectra of **PP1**, **PP2** and their metal conjugates (Figures S5 and S6) are generally similar but with differing maxima. The spectra of the metalloporphyrins contain less intense emission bands that are blue-shifted compared to those of the free bases. The Φ*_F_* values for the metalloporphyrins are lower than those of **PP1** and **PP2** (Table 2), due to the heavy atom ensuring strong spin-orbit coupling and enhanced intersystem crossing (ISC) [47,48,49]. Improved ISC populates the triplet states lowering fluorescence quantum yield. The Φ_F_ values of ~0.01 for **InPP1** and **InPP2** are the lowest. The τ_F_ values for the metalloporphyrins were shorter than those of free base porphyrins (Table 2) due to the heavy atom effect.

### 2.3. Density Function Theory Calculations

**Figure 4:**
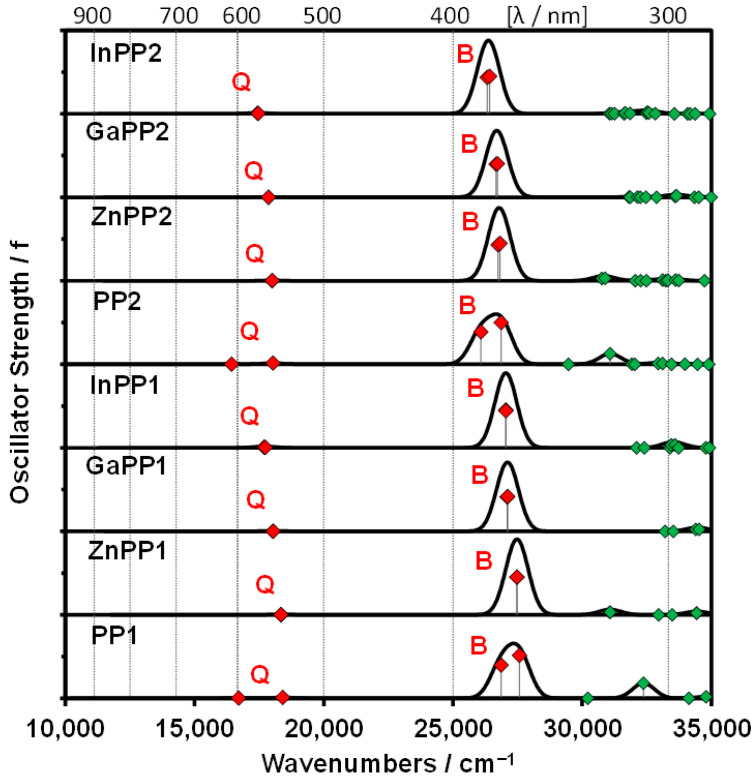
TD-DFT spectra for **PP1**, **PP2,** and the metal complexes using the CAM-B3LYP functional with either SDD or 6-31G(d) basis sets with green diamonds highlighting the oscillator strength values for bands predicted to lie below 35,000 cm^−1^. Large red diamonds are used to highlight the Q and B bands of Gouterman’s 4-orbital model.^33^ Simulation of the spectra was carried out using the Chemcraft software package, Version 1.8 (build 536b) with fixed bandwiths of 1000 cm^−1^. Details of the calculations are provided in Table 3.

Density functional theory (DFT) computations were performed to help identify suitable compounds for photosensitization and compute the photophysical properties of the synthesized porphyrins for comparison with experimental values. The spectral bands observed in the visible region of the absorption spectra of **PP1**, **PP2** and their metal complexes can be readily assigned to the forbidden Q and allowed B transitions of Gouterman’s 4-orbital model on the basis of the TD-DFT calculations (Figure 4 and Table 3). Only the Q and B bands are predicted to lie in the visible region when a systematic over-estimation of the band energies is taken into account, since other π-MOs are well separated in energy terms from the **a**, **s**, **−a,** and **−s** MOs (Figure 3). The calculated Q and B band maxima for *meso*-5-bromothien-2-yl-substituted **PP2**, **ZnPP2**, **GaPP2,** and **InPP2** are red shifted compared to those of *meso*-phenyl-substituted **PP1**, **ZnPP1**, **GaPP1**, and **InPP1** (Figure 4 and Table 3), thus exhibiting the same trend observed in the experimental values (Figure 1 and Figure 2, Table 1). The major spectral bands of **InPP1** and **InPP2** are more red shifted than those of Zn(II) and Ga(III) complexes (Table 3). This is also consistent with the trend observed experimentally (Figure 1 and Figure 2, Table 1) due to a narrowing of the HOMO−LUMO gap (Figure 3). A relative destabilization of the **s** MOs of **InPP1** and **InPP2** is predicted, which has very large MO coefficients at the *meso*-carbons (Figure 3), while there is a large stabilization of the **a**, **−a,** and **−s** MOs due to the electron-withdrawing effect of the trivalent central In(III) ion.

### 2.4. Suitability for Electron Injection

Figure 3 compares the relative HOMO and LUMO energies of the **PPs** studied here with the energies of the valence and conduction band (VB and CB respectively) edges of TiO_2_. It can be observed from the DFT calculations (Figure 3) that the HOMO and LUMO energy levels of the photosensitizers fit the requirements for an efficient photosensitizer [50], since the LUMO energies of the photosensitizers lie between −2.48 and −3.34 eV, well above the TiO_2_ CB (ca. −4.0 eV), implying that efficient electron injection from the photoexcited sensitizer into the TiO_2_ CB should be viable [51]. It was anticipated that the stabilization of the frontier π-MOs associated with the incorporation of Ga(III) and In(III) ions (Figure 3) would better match the TiO_2_ CB due to the electron-withdrawing effect of the trivalent ions and that this would be further enhanced by the electron-withdrawing properties of the *meso*-5-bromothien-2-yl rings of **PP2**.

### 2.5. Characterization of the Porphyrin-TiO_2_ (P-TiO_2_) Photocatalysts by Diffuse Reflectance Spectroscopy

**Figure 5:**
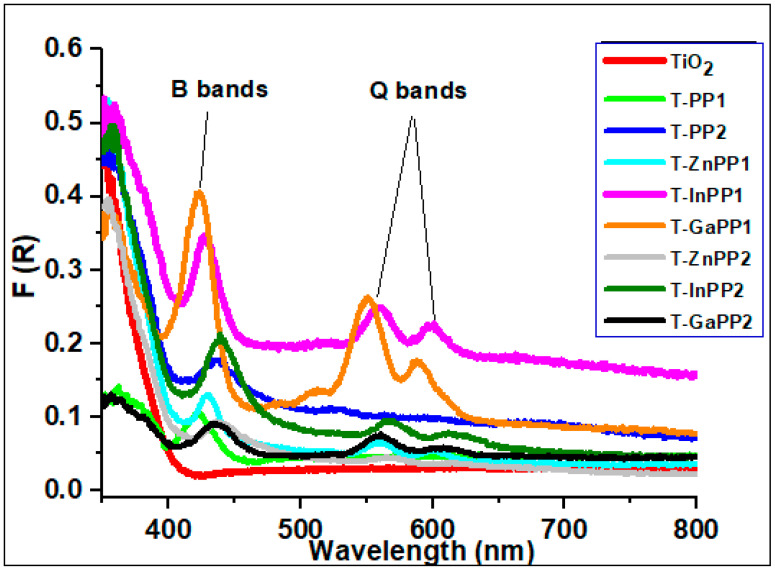
Diffuse Reflectance spectra of the **P**-TiO_2_ photocatalysts.

UV-visible diffuse reflectance spectra (Figure 5) provide evidence for the presence of porphyrins on the TiO_2_ surface. TiO_2_ does not exhibit any absorbance in the visible region at >400 nm, while the spectra of the **P**-TiO_2_ photocatalysts contain the characteristic B and Q bands of the porphyrins. The presence of these bands demonstrates that porphyrins were successfully loaded onto the surface of TiO_2_. The photocatalysts are therefore anticipated to exhibit a significantly broader absorption range. To infer the amount of porphyrin loaded in each system, the intensity of the porphyrins Soret bands were compared using the normalized diffuse reflectance spectra (Figures S7 and S8). The analyses suggest that in general **PP1** porphyrins attach better to the surface of TiO_2_ than their PP2 counter parts. Within the both **PP1** and **PP2** series, the attachment follows the trend **G**a-**P**P > **PP~In-PP** >> **Zn-PP**.

### 2.6. Photocatalytic Activity of the Photocatalysts

**Figure 6:**
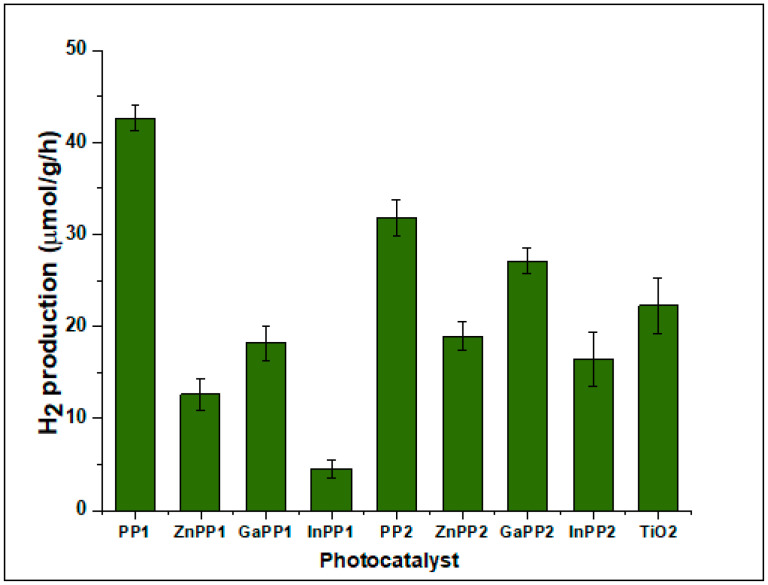
Hydrogen generation rates for the **P**-TiO_2_ photocatalysts in 1% methanol solution under simulated solar light.

The activity of the **P**-TiO_2_ photocatalysts in the generation of hydrogen in aqueous solution was evaluated for each of the photocatalysts. Eight photocatalysts, namely: **ZnPP1**-TiO_2_, **GaPP1**-TiO_2_, **GaPP2**-TiO_2_, **InPP2**-TiO_2_ **ZnPP2**-TiO_2_, **InPP1**-TiO_2_, **PP1**-TiO_2_, and **PP2**-TiO_2_, were assessed in 1% methanol solution. All the photocatalysts exhibited the ability to generate H_2_ (Figure 6), including bare TiO_2_. **PP1**-TiO_2_, **PP2**-TiO_2,_ and **GaPP2**-TiO_2_ showed a superior photocatalytic activity rate for H_2_ generation relative to bare TiO_2_. In the case of native **PPs**, H_2_ production from **PP1** is at least 30% higher than that found for **PP2.** Although this could be related to slightly higher **PP1** loading, differences in the porphyrin loadings cannot explain the decrease of in production of H_2_ when using metalloporphyrins, particularly **Ga-PP** and **In-PP** that show higher to similar loadings compared to their native **PPs**. Therefore, the variation in the activity of the systems could be a result of different electron dynamics between the surface of the dye and TiO_2_. Freebase porphyrins (**PP1** and **PP2**) had better photosensitization than their metal complexes due to more electronic coupling with the TiO_2_ surface. Both **PP1** and **PP2** being metal free are flexible with fairly non-planar conformations thus can afford multiple adsorption configurations due to variable dihedral angles. The freebase cavity can co-ordinate more effectively with TiO_2_ by flat attachment favoring electron injection in comparison to the metal complexes [52]. Additionally, metallophorphyrins have greater non-radiative decays compared to their native porphyrins suggesting fast singlet state decay, which can hamper electron injection into TiO_2_.

### 2.7. Mechanism of Photocatalysis

**Scheme 2:**
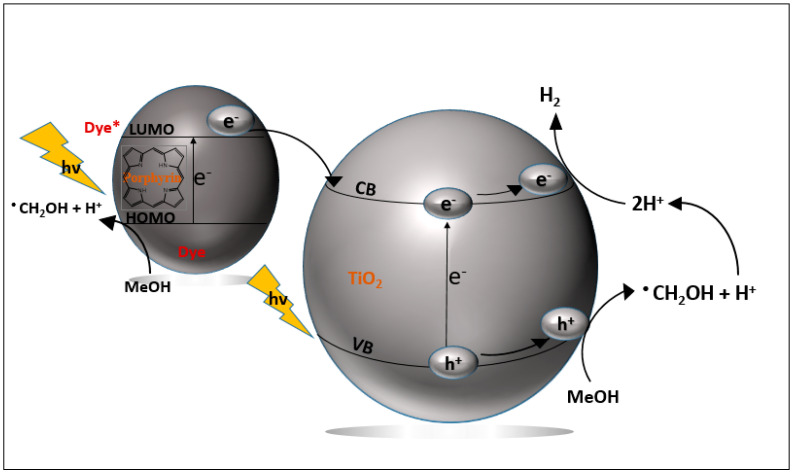
Proposed mechanism for H_2_ generation by TiO_2_ on sensitization with porphyrins [53,54,55].

The proposed mechanism for the photochemical reactions of the sacrificial molecules is illustrated in Scheme 2. Methanol was used as a sacrificial electron donor to facilitate the generation of H_2_, for simplicity Scheme 2 shows only the first oxidation step (formation of hydroxymethyl radical) although further oxidation is expected [53]. Under simulated solar irradiation, UV and visible light energy can be absorbed by the **P**-TiO_2_ composite. In addition to the direct excitation of TiO_2_ creating electron-hole pairs, injection of electrons from the LUMO of photoexcited porphyrins into the TiO_2_ conduction band is also energetically favorable (Figure 3) for all **P**-TiO_2_ systems studied. This could generate a continuous flux of electrons into the TiO_2_ CB that are readily available for the reduction of the H^+^ ion. Significantly, more efficient H_2_ production was obtained when **PP1**-TiO_2_ and **PP2**-TiO_2_ composites were used. With the exception of **GaPP2**-TiO_2_, the composites prepared from metal porphyrin complexes have lower H_2_ production efficiencies than that obtained for bare TiO_2_. This could be related to the ability of the heavy metals to favor ISC and hence populate triplet states (laying at lower energy levels). This is likely to hamper rather than enhancing the electron flow from the LUMO of the photoexcited porphyrin to the TiO_2_ CB. The presence of the heavy sulfur atoms of the *meso*-aryl rings of **PP2** could explain why the photocatalytic properties of the **PP1**-TiO_2_ composite were superior to those of **PP2**-TiO_2_. Interestingly, however, the **MPP2**-TiO_2_ composites are consistently more efficient than the analogous **MPP1**-TiO_2_ materials. This is consistent with the higher Φ_F_ and τ_F_ values for these complexes in Table 2, which is consistent with greater population of the S_1_ state that can be expected to enhance electron injection from the photoexcited sensitizer into the TiO_2_ CB. In a similar manner, the Φ_F_ and τ_F_ values for **PP1** are higher than those for **PP2** (Table 2). It is noteworthy that although the **InPP1** and **InPP2** are predicted to lie closer in energy to the TiO_2_ CB (Figure 3), it is the **GaPP1**-TiO_2_ and **GaPP2**-TiO_2_ materials that have superior photocatalytic effects (Figure 6) among the metallophorphyrins studied.

## 3. Materials and Methods

### 3.1. Materials

Pyrrole, 4-bromobenzaldehyde, dichloromethane (DCM), methanol (MeOH), high purity grade silica gel (35–60 mesh particle size, thin layer chromatography (TLC) plates, sodium acetate, 5-bromo-2-thiophenecarboxaldehyde, indium(III) chloride, tetraphenylporphyrin (H_2_TPP), zinc tetraphenylporphyrin (ZnTPP) and gallium (III) chloride were purchased from Sigma Aldrich (Munich, Germany). Hexane, propionic acid, sodium hydrogen carbonate, acetic acid and zinc acetate were purchased from Loba Chemie PVT Ltd, Mumbai, India. Toluene and ethyl acetate were purchased from Fisher Scientific, Leicestershire, UK. Distilled water was obtained from an ultrapure filtration system (Millipore, resistivity 18 mΩ cm^−1^). TiO_2_ P25 was purchased from Univar Canada.

### 3.2. Instrumentation

The ground state electronic absorption spectra were obtained at room temperature from an Agilent Technologies Cary 60 UV-Vis spectrophotometer in 1.0 × 1.0 cm quartz cuvettes, while corrected emission spectra were obtained with a PTI Quanta Master spectrofluorimeter (Photon Technology International, of London, ON, Canada) and 0.4 × 1.0 cm cuvettes. The IR spectra were recorded on a Nicolet 6700 FT-IR spectrometer (Madison, WI, USA), and centrifugation was carried out on an Eppendorf centrifuge 5804 R. Fluorescence lifetimes were measured using a time-correlated EasyLife fluorescence lifetime fluorimeter (Photon Technology International, of London, ON, Canada). The instrument response function (IRF) was measured at the excitation wavelength (395 nm) using Ludox as the scatterer. Mass spectrometry data were obtained from a Bruker AutoFLEX III Smartbeam MALDI-TOF instrument (Billerica, MA, USA). The elemental analyses were carried out on a Vario EL III Microcube CHNS Analyzer. H NMR spectra were obtained from a Bruker AVANCE II 400 MHz NMR spectrometer (Burlinton, Ontario, Canada). with CDCl_3_ as the solvent. Diffuse reflectance spectroscopy (DR) data were collected on a Varian Cary 100 UV-Vis spectrophotometer (Santa Clara, CA, USA) coupled to an integrating sphere accessory. Samples were irradiated using a Luzchem SolSim CCP instrument (Ottawa, Canada) providing simulated solar radiation approximately matching in intensity the 1.5 air mass (AM) solar spectrum. The hydrogen generated was detected and quantified with a Perkin Elmer, Clarus 480 GC-TCD (Madison, WI, USA) using argon as a carrier gas and a 5 A zeolite molecular sieve column.

### 3.3. Synthesis of Porphyrins

The aromatic *meso*-substituted porphyrins **PP1** and **PP2** were synthesized by a single step acid-catalyzed condensation reaction of pyrrole with an appropriate aldehyde based on the Adler-Longo method [56,57]. Their metal complexes were synthesized according to literature methods [58,59]. Scheme 1 gives a summary of the synthesis procedure used and the reaction conditions.

### 3.4. Photophysical Parameters

As illustrated in Equation (1), comparative methods were used to determine fluorescence quantum yield (Φ_F_) values. Tetraphenylporphyrin (H_2_TPP) in toluene was used as a standard ($\Phi_{F}^{\mathrm{Std}}$ = 0.11) for the metal-free porphyrins (**PP1** and **PP2**), while zinc tetraphenylporphyrin (ZnTPP) in toluene was the standard ($\Phi_{F}^{\mathrm{Std}}$ = 0.033) for the metalated porphyrins [60].

(1) $$\Phi_{F}=\frac{{F\;A}^{\mathrm{Std}}{\;n}^{2}}{F^{\mathrm{Std}}A\;{\left(n^{\mathrm{Std}}\right)}^{2}}{\;\Phi }_{F}^{\mathrm{Std}}$$

In this equation, A and A^Std^ are the optical densities for the sample and standard solutions respectively, while F and F^Std^ are the integrated areas under the emission curves for sample and standard, n and n^Std^ are the refractive indices of the solvents. The radiative (k_r_) and non-radiative (k_nr_) rate constant data were obtained by calculation according to Equations (2) to (3) [61].

(2) $$\Sigma \;k\;=\frac{1}{\tau_{F}}$$

(3) $$k_{r}={\;\Phi }_{F}\times \Sigma \;k$$

(4) $$k_{\mathrm{nr}}\;=\;\Sigma \;k\;-{\;k}_{r}$$

Fluorescence lifetime (τ_F_) values were determined using a time-correlated EasyLife X filter fluorescence lifetime fluorimeter. Time-resolved fluorescence decay curves were analyzed by deconvoluting the observed decay with the IRF to obtain the intensity decay function. Reduced chi-squared values were evaluated and considered to determine the best fit for experimental data.

### 3.5. Theoretical Calculations

Quantum calculations were performed using the Gaussian 09 program [62] and the theoretical model of choice was density functional theory (DFT). DFT is useful in providing direct information on electronic structure and strikes a favorable balance between computational cost and accuracy [63]. The gas-phase geometries of the porphyrin monomer structures were optimized by Becke’s 3-parameter Lee Yang Parr (B3LYP) exchange-correlation functional. Linear time-dependent DFT (TD-DFT) calculations were performed with the Coulomb Attenuating Method B3LYP (CAM-B3LYP) functional. Mixed basis sets were used for these calculations; 6-31G(d,p) and Stuttgart-Dresden (SDD), the latter being preferred for porphyrin monomers with In(III) or Ga(III) metal ions. Simulation of the spectra was carried out using the Chemcraft software package with fixed bandwidths of 1000 cm^−1^.

### 3.6. Supporting Porphyrins on TiO_2_ P25

A procedure reported by Duan and co-workers in 2010 [64] was followed to deposit the porphyrins on the surface of TiO_2_. The porphyrins (10 mg) were dissolved in DCM (8 mL) in eight separate vessels. To each solution, TiO_2_ (0.4 g) was added, and the mixture was sealed. The resulting suspension was sonicated for 30 min, then stirred at ambient temperature for 12 h. The photocatalyst was collected by centrifugation after washing three times with DCM and left to dry at 60 °C for 12 h.

### 3.7. Photocatalytic Hydrogen Generation

Photocatalytic hydrogen generation studies were carried out according to the procedure initially used by Hainer et al. [11]. These studies were carried out in a 10 mL glass vial sealed with crimp seals and rubber septa. In each experiment, 10 mg of the photocatalyst and 4 mL aqueous methanol solution (1%) was added to the glass vial. The system was degassed under argon for 30 min and sealed in the vacuum degassing chamber. The catalyst suspension was dispersed for 5 min in an ultrasonic bath then irradiated from the top for 4 h under simulated solar light with magnetic stirring. Headspace gas (4 mL) was sampled from each vial by a sample lock syringe through the septum and immediately injected in the GC-TCD. The H_2_ signal was detected at ~4.0 min. A calibration curve for H_2_ gas detection in the GC-TCD instrument was used for quantification.

## 4. Conclusions

In conclusion, *meso*-tetra(4-bromophenyl)porphyrin **PP1** and *meso*-tetra(5-bromo-2-thienyl)porphyrin **PP2** and their Zn(II), (Cl)In(III) and (Cl)Ga(III) complexes were successfully synthesized and characterized. Diffuse reflectance spectroscopy demonstrated that the porphyrins were successfully loaded onto the surface of TiO_2_ P25. **PP2**-TiO_2_, **PP1**-TiO_2_ and **GaPP2**-TiO_2_ exhibited superior hydrogen generation compared to bare TiO_2_ demonstrating their potential utility for photocatalytic H_2_ generation. A wide range of porphyrin analogs exist with enhanced absorption in the Q band region, so research will continue in identifying dyes that are best suited for this application by focusing on dyes with long-lived and well-populated S_1_ excited states.

## Figures and Tables

**Table 1:** The UV-visible absorption data for **PP1**, **PP2** and their metal complexes in toluene. Please note: the assignment of the Q bands (Q_yo1_, Qy_00_, Q_xo1_, Q_x00_) is shown in Figure 2.

	**Soret Band (nm)** **(ε × 10^5^) (L mol^−1^ cm^−1^)**	**Q Bands (nm) (ε × 10^3^) (L mol^−1^ cm^−1^)**			
		**Q_y01_**	**Q_y00_**	**Q_x01_**	**Q_x00_**
**H_2_TPP**	421	515	549	591	649
**PP1**	421 (2.1)	517 (9.4)	550 (4.5)	593 (2.7)	650 (2.1)
**PP2**	431 (2.6)	524 (13.5)	561 (7.0)	600 (4.5)	665 (3.7)
	**Soret Band (nm)** **(ε × 10^5^) (L mol^−1^ cm^−1^)**	**Q Bands (nm) (ε × 10^3^) (L mol^−1^ cm^−1^)**			
		**Q_01_**		**Q_00_**	
**ZnPP1**	425 (9.6)	550 (52.8)		589 (10.6)	
**InPP1**	430 (5.4)	563 (31.0)		602 (13.2)	
**GaPP1**	424 (6.9)	553 (29.0)		592 (6.2)	
**ZnPP2**	433 (8.0)	559 (51.9)		602 (8.5)	
**InPP2**	439 (3.0)	570 (15.7)		613 (6.6)	
**GaPP2**	434 (3.9)	561 (22.7)		604 (4.7)	

**Table 2:** Photophysical parameters of **PP1**, **PP2** and their metal complexes in toluene. Q_yo1_, Qy_00_, Q_xo1_, Q_x00_) is shown in Figure 2. The equations for the determination of fluorescence quantum yield (Φ_F_) and lifetime (τF), the radiative (k_r_) and non-radiative (k_nr_) rate constant are provided in Section 3.4.

	λ_em_ (nm)		Stokes Shift cm^−1^	Φ_F_	$\tau_{F}\;(\mathrm{ns})$	$k_{r}$ (10^8^ s^−1^)	$k_{nr}$ (10^8^ s^−1^)
	$Q_{00}$	$Q_{01}$					
**PP1**	654	717 (sh)	8460	0.019	1.55 ± 0.02	0.123	6.33
**ZnPP1**	600 (sh)	649	8120	0.010	0.38 ± 0.01	0.255	26.1
**GaPP1**	597 (sh)	650	8200	0.013	0.48 ± 0.01	0.269	20.6
**InPP1**	610 (sh)	660	8100	0.006	0.24 ± 0.02	0.233	41.4
**PP2**	670	730 (sh)	8280	0.017	1.37 ± 0.02	0.124	7.18
**ZnPP2**	621 (sh)	659	7920	0.012	0.54 ± 0.01	0.219	18.3
**GaPP2**	624 (sh)	663	7960	0.013	0.53 ± 0.01	0.240	18.6
**InPP2**	633	673 (sh)	6980	0.010	0.29 ± 0.02	0.331	34.2

**Table 3:** Electronic excitation bands of the synthesized porphyrins at the TD-DFT level of theory using the CAM-B3LYP functional with either SDD or 6-31G(d) basis sets.

	Band ^a^	# ^b^	Calc. ^c^			Exp. ^d^		Wave Function ^e^
**PP1**	Q	1	16.7	599	0.01	15.4	650	59% **s → −s;** 40% **a → −a;** …
	Q	2	18.4	543	0.04	17.1	586	59% **s → −a;** 40% **a → −s;** …
	B	3	26.9	372	1.44	23.7	421	57% **a → −a;** 32% **s → −s;** …
	B	4	27.6	363	1.85			58% **a → −s;** 39% **s → −a;** …
**ZnPP1**	Q	1, 2	18.3	545	0.01	17.0	587	53% **s → −a/−s;** 46% **a → −a/−s;** …
	B	3, 4	27.5	364	1.63	23.5	425	46% **a → −a/−s;** 44% **s → −a/−s;** …
**GaPP1**	Q	1, 2	18.0	554	0.01	17.0	589	50% **s → −a/−s;** 41% **a → −a/−s;** …
	B	3, 4	27.1	368	1.48	23.6	424	55% **a → −a/−s;** 42% **s → −a/−s;** …
**InPP1**	Q	1, 2	17.7	565	0.03	16.6	601	60% **s → −a/−s;** 39% **a → −a/−s;** …
	B	3, 4	27.0	370	1.63	23.3	430	58% **a → −a/−s;** 37% **s → −a/−s;** …
**PP2**	Q	1	16.4	609	0.01	15.0	665	57% **s → −s;** 42% **a → −a;** …
	Q	2	18.0	555	0.06	16.6	602	59% **s → −a;** 40% **a → −s;** …
	B	3	26.1	383	1.40	23.2	431	54% **a → −a;** 34% **s → −s;** …
	B	4	26.9	372	1.80			58% **a → −s;** 38% **s → −a;** …
**ZnPP2**	Q	1, 2	18.0	556	0.01	16.6	602	50% **s → −a/−s;** 48% **a → −a/−s;** …
	B	3, 4	26.7	374	1.54	23.1	433	49% **a → −a/−s;** 44% **s → −a/−s;** …
**GaPP2**	Q	1, 2	17.9	560	0.00	16.6	603	50% **s → −a/−s;** 49% **a → −a/−s;** …
	B	3, 4	26.7	375	1.43	23.0	434	49% **a → −a/−s;** 48% **s → −a/−s;** …
**InPP2**	Q	1, 2	17.4	573	0.02	16.2	616	55% **s → −a/−s;** 43% **a → −a/−s;** …
	B	3, 4	26.3	380	1.57	22.8	439	53% **a → −a/−s;** 40% **s → −a/−s;** …

**^a^** Band assignment referred to in this work. **^b^** Energy states assigned in ascending order within the TD-DFT calculation. **^c^** Calculated (Calc) band energies (10^3^ cm^−1^), wavelengths (nm) and oscillator strengths in brackets (f). **^d^** Experimental (Exp) energies (10^3^ cm^−1^) and wavelengths (nm), **^e^** The wave functions based on the eigenvectors predicted by TD-DFT. One-electron transitions between the **a**, **s**, **−a** and **−s** MOs are highlighted in bold. Only contributions greater than 10% are provided.

## Data Availability

Data is contained within the article or Supplementary Materials.

## Supplementary Materials

The following are available online: details for the synthesis and characterization of the porphyrins as well as their 1H NMR, mass, fluorescence spectra and Diffuse reflectance spectra (Figures S1–S8).

## Sample Availability

Samples of the compounds are available from the authors.
