# Supplementary material for: Solar Driven Photocatalytic Activity of Porphyrin Sensitized TiO2: Experimental and Computational Studies
Source: Molecules. 2021 May 24;26(11):3131. doi: 10.3390/molecules26113131 (PMC8197238; doi:10.3390/molecules26113131)
Supplement: Supplementary file 1 [file molecules-26-03131-s001.zip › molecules-1174383-supplementary.pdf]

Solar driven photocatalytic activity of porphyrin sensitized TiO_2: experimental and computational studies

Sebastian Otieno[a,b,c], Anabel E. Lanterna[d†], John Mack[e], Solomon Derese[c], Edith K. Amuhaya[b*], Tebello Nyokong[e*], Juan C. Scaiano[a*]

[a] Department of Chemistry and Biomolecular Sciences and Centre for Advanced Materials Research (CAMaR), University of Ottawa, 10 Marie Curie, Ottawa, Ontario K1N 6N5, Canada

[b] School of Pharmacy and Health Sciences, United States International University - Africa, Nairobi, Kenya

[c] Department of Chemistry, University of Nairobi, Nairobi, Kenya

[†d] School of Chemistry, University of Nottingham, University Park, Nottingham, NG7 2RD, UK

[e]Department of Chemistry, Rhodes University, South Africa

Experimental Methods **2**

1. *Synthesis of meso-tetra (4-bromophenyl)porphyrin (**PP1**)* 2
2. *Synthesis of meso-tetrakis (5-bromo-2-thiophenyl)porphyrin (**PP2**)* 2
3. *Synthesis of Zn, ClIn and ClGa metal conjugates of **PP1** and **PP2*** 3

Characterization of PP1, PP2 and their metal complexes **5**

Figure S1: ^{1}H NMR spectrum for **PP1** 6

Figure S2: ^{1}H NMR spectrum for **PP2** 7

Figure S3: Mass spectrum for **PP1**. 7

Figure S 4: Mass spectrum for **PP2**. 8

Figure S 5: Fluorescence spectra of **PP1** and its metal complexes in toluene. 8

Figure S 6: Fluorescence spectra of **PP2** and its metal complexes in toluene. 9

Figure S 7: Normalized Diffuse Reflectance spectra for **TiO_2** on **PP1** and its metal complexes. 9

Figure S 8: Normalized Diffuse Reflectance spectra for **TiO_2** on **PP2** and its metal complexes. 10

Experimental Methods

1. *Synthesis of meso*-tetra *(4-bromophenyl)porphyrin (**PP1**)*

The synthesis was carried out according to literature methods with slight modifications as follows: Freshly distilled pyrrole (1.20 mL, 18 mmol) was added dropwise to a warm solution of 4-bromobenzaldehyde (3.24 g, 18 mmol) in propionic acid (80 mL). The mixture was brought to reflux temperature (125−130°C) with stirring for 3 h. The progress of the reaction was continually monitored by TLC and UV-visible absorption spectroscopy. The reaction vessel was allowed to cool to room temperature and 50 mL methanol was added to further quench the solution and aid crystallization. The product was collected as a purple solid by vacuum filtration, washed with methanol and left to dry in open air. The product was further purified by elution in a column of silica gel with ethyl acetate: hexane (1:9 v/v) mobile phase.

Yield: Purple solid 2.01 g (12%). UV-vis (Toluene) λ_{max} nm (ε (L Mol^{-1} cm^{-1})): 421 (2.1×10^5), 517 (9.4×10^3), 550 (4.5×10^3), 593 (2.7×10^3) and 650 (2.1×10^3). ^{1}H NMR (400 MHz, $CDCl_3$) δ_H ppm 8.84 (*s*, 8H, β Pyrrol), 8.06 (*d*, *J*= 8.3 Hz, 8H), 7.90 *(d*, *J*= 8.3 Hz, 8H). IR ν_{max} cm^{-1} 3320 (N-H), 3000 (=C-H_{str}), 1370 (C-N_{str}). Calc. for $C_{44}H_{26}Br_4N_4$: C = 56.81, H = 3.03, N = 6.01; found C = 56.54, H = 3.00, N = 6.10. MALDI-TOF-MS m/z: calcd = 930.34, found = 930.94 [M^+].

2. *Synthesis of meso-tetrakis (5-bromo-2-thiophenyl)porphyrin (**PP2**)*

PP2 was prepared in the same manner as **PP1**. Freshly distilled pyrrole (1.04 mL, 15 mmol) and 5-bromo-2-thiophenecarboxaldehyde (1.78 mL, 15 mmol) were refluxed for 30 min.

Yield: Purple solid 1.43 g (10%). UV-vis (Toluene) λ_{max} nm (ε (L Mol^{-1} cm^{-1})): 431 (2.6×10^5), 524 (13.5×10^3), 561 (7.0×10^3), 600 (4.5×10^3), 665 (3.7×10^3). ^{1}H NMR (400 MHz, $CDCl_3$) δ_H ppm 9.11 (s, 8H, β Pyrrol), 7.66 (*d, J*= 3.6 Hz, 4H), 7.49 (*d, J*= 6.35 Hz, 4H). IR ν_{max} cm^{-1} 3310 (N-H), 3100 (=C-Hstr), 1250 (C-Nstr). Calc. for $C_{36}H_{18}Br_4N_4S_4$: C = 45.30, H = 1.90, N = 5.87, S = 13.44; found C = 45.67, H = 1.80, N = 5.61, S = 13.46. MALDI-TOF-MS m/z: calcd = 954.42, found = 954.78 [M^+].

*3. Synthesis of Zn, ClIn and ClGa metal conjugates of **PP1** and **PP2***

The indium and gallium metalloporphyrins were synthesized as follows: To warm acetic acid (50 mL), sodium acetate, free base porphyrin (**PP1** or **PP2**) and either gallium or indium chloride was added and the solution brought to reflux temperature (115−119 °C). With stirring, either of the solutions were refluxed for 15 and 36 h for gallium and indium respectively. The progress of the reaction was monitored by TLC and UV-visible absorption spectroscopy. Acetic acid was removed in *vacuo* and sodium hydrogen carbonate was added to neutralize remaining acid. The product was dissolved in DCM and washed three times in a separatory funnel with distilled water. The product was retrieved from DCM under vacuum and further purified by column chromatography.

Reagents used for **GaPP1** were **PP1** (0.60 g, 0.645 mmol), gallium chloride (0.36 g, 2.096 mmol) and sodium acetate (0.44 g, 3.225 mmol). The product was purified over silica gel column using methanol: dichloromethane (2:8 v/v) mobile phase.

Yield: Purple solid 0.43 g (65%). UV-vis (Toluene) λ_{max} nm (ε (L Mol^{-1} cm^{-1})): 424 (6.9×10^5), 553 (29×10^3), 592 (6.2×10^3). ^{1}H NMR (400 MHz, $CDCl_3$) δ_H ppm 8.82−9.06 (m, 8H, β Pyrrol), 8.04 (*d, J*= 8.3 Hz, 8H), 7.89 (*d, J*= 8.5 Hz, 8H). IR ν_{max} cm^{-1} 2900 (=C-H_{str}), 1250 (C-N_{str}). Calc. for $C_{44}H_{24}Br_4ClGaN_4$: C = 51.14, H = 2.34, N = 5.42; found C = 51.64, H = 2.39, N = 5.33. MALDI-TOF-MS m/z: calcd = 1033.49, found = 997.97 [M-Cl]$^+$.

Reagents used for **GaPP2** were **PP2** (0.40 g, 0.419 mmol), gallium chloride (0.23 g, 1.341 mmol) and sodium acetate (0.29 g, 2.096 mmol). The product was purified over silica gel column using methanol: dichloromethane (2:8 v/v) mobile phase.

Yield: Purple solid 0.27 g (61%). UV-vis (Toluene) λ_{max} nm (ε (L Mol^{-1} cm^{-1})): 434 (3.9×10^5), 561 (22.7×10^3), 604 (4.7×10^3). ^{1}H NMR (400 MHz, $CDCl_3$) δ_H ppm 9.35 (s, 8H, β Pyrrol), 7.68 (*d, J*= 3.7 Hz, 4H), 7.48 (*d, J*= 7.2 Hz, 4H). IR ν_{max} cm^{-1} 2900 (=C-Hstr), 1380 (C-Nstr). Calc. for $C_{36}H_{16}Br_4ClGaN_4S_4$: C = 40.89, H = 1.52, N = 5.30, S = 12.13; found C = 40.72, H = 1.58, N = 5.32, S = 12.02. MALDI-TOF-MS m/z: calcd = 1057.58, found = 1022.77 [M-Cl]$^+$.

Reagents used for **InPP1** were **PP1** (0.60 g, 0.645 mmol), indium chloride (0.29 g, 1.230 mmol) and sodium acetate (0.44 g, 3.225 mmol). The product was purified over silica gel column using ethyl acetate: hexane (1:9 v/v) solvent system.

Yield: Purple solid 0.38 g (55%). UV-vis (Toluene) λ_{max} nm (ε (L Mol^{-1} cm^{-1})): 430 (5.4×10^5), 563 (31×10^3), 602 (13.2×10^3). ^{1}H NMR (400 MHz, $CDCl_3$) δ_H ppm 9.02 (s, 8H, β Pyrrol), 8.23 (*d, J*=8.5 Hz, 4H), 7.88–7.89 (*m*, 12H,). IR ν_{max} cm^{-1} 2900 (=C-H_{str}), 1250 (C-N_{str}). Calc. for $C_{44}H_{24}Br_4ClInN_4$: C = 49.00, H = 2.24, N = 5.19; found C = 48.36, H = 2.38, N = 5.47. MALDI-TOF-MS m/z: calcd = 1078.59, found = 1043.38 $[M\text{-}Cl]^+$.

Reagents used for **InPP2** were **PP2** (0.40 g, 0.419 mmol), indium chloride (0.19 g, 0.838 mmol) and sodium acetate (0.29 g, 2.096 mmol). The product was purified over silica gel column using ethyl acetate: hexane (1:9 v/v) solvent system.

Yield: Purple solid 0.23 g (49%). UV-vis (Toluene) λ_{max} nm (ε (L Mol^{-1} cm^{-1})): 439 (3.0×10^5), 570 (15.7×10^3), 613 (6.6×10^3). ^{1}H NMR (400 MHz, $CDCl_3$) δ_H ppm 9.33 (s, 8H, β Pyrrol), 7.70 (*d, J*= 4.6 Hz, 4H), 7.50 (*d, J*= 4.6 Hz, 4H). IR ν_{max} cm^{-1} 2900 (=C-Hstr), 1250 (C-Nstr). Calc. for $C_{36}H_{16}Br_4ClInN_4S_4$: C = 39.21, H = 1.46, N = 5.08, S = 11.63; found C = 39.50, H = 1.38, N = 5.11, S = 12.01. MALDI-TOF-MS m/z: calcd = 1102.68, found = 1067.79 $[M\text{-}Cl]^+$.

Zinc metalloporphyrins were synthesized as follows: To a solution of free base porphyrin (**PP1** or **PP2**) a saturated solution of zinc acetate in methanol was added and the mixture was stirred at rt for 12 h. The solvent was removed in vacuo and the product was purified.

Reagents used for **ZnPP1** were **PP1** (0.30 g, 0.323 mmol), zinc acetate (0.24 g, 1.093 mmol), methanol (15 mL) and DCM (45 mL). The product was purified over silica gel column using ethyl acetate: hexane (1:9 v/v) mobile phase.

Yield: Purple solid 0.27 g (83%). UV-vis (Toluene) λ_{max} nm (ε (L Mol^{-1} cm^{-1})): 425 (9.6×10^5), 550 (52.8×10^3), 589 (10.6×10^3). ^{1}H NMR (400 MHz, $CDCl_3$) δ_H ppm 8.94 (s, 8H, β Pyrrol), 8.07 (*d, J*= 8.4 Hz, 8H), 7.90 (*d, J*= 8.4 Hz, 8H). IR ν_{max} cm^{-1} 2900 (=C-H_{str}), 1270 (C-N_{str}). Calc. for $C_{44}H_{24}Br_4ZnN_4$: C = 53.18, H 2.43, N 5.64; found C = 53.55, H 2.75, N 5.52.

Reagents used for **ZnPP2** were **PP2** (0.25 g, 0.262 mmol), zinc acetate (0.17 g, 0.786 mmol), methanol (15 mL) and DCM (45 mL). The product was purified over silica gel column using ethyl acetate: hexane (1:9 v/v) solvent system.

Yield: Purple solid 0.21 g (78%). UV-vis (Toluene) λ_{max} nm (ε (L Mol^{-1} cm^{-1})): 433 (8.0×10^5), 559 (51.9×10^3), 602 (8.5×10^3). ^{1}H NMR (400 MHz, $CDCl_3$) δ_H ppm 9.19 (s, 8H, β Pyrrol), 7.63 (*d, J*= 6.2 Hz, 4H), 7.46 (*d, J*= 3.6 Hz, 4H). IR ν_{max} cm^{-1} 2900 (=C-Hstr), 1250 (C-Nstr). Calc. for $C_{36}H_{16}Br_4ZnN_4S_4$: C = 42.48, H = 1.58, N = 5.50, S = 12.60; found C = 42.37, H 1.61, N 5.43, S 12.52.

Characterization of PP1, PP2 and their metal complexes

Mass spectrometry, ^{1}H NMR, FTIR spectra, and elemental analysis were used to characterize the porphyrins. Results obtained were satisfactory and consistent with the proposed structures.

The ^{1}H NMR spectrum for **PP1** (Fig. S1) contains a singlet at δ_H 8.84 ppm integrating for eight protons and is assigned for the β-protons of the four pyrrole rings. These protons at the β position lie outside the macrocyclic ring current thus experience a deshielding effect raising the chemical shift to higher values. The ^{1}H NMR further showed signals for an AA'BB' spin system centred at δ_H 8.07 and 7.90 (*d*, *J* = 8.3 Hz) ppm integrating for sixteen protons representing the four *meso*-substituted aromatic rings. For **PP2**, the ^{1}H NMR (Fig. S2) showed a singlet at δ_H 9.11 ppm integrating for eight protons and was assigned to the β-protons of the four pyrrole rings. As is the case with **PP1**, these protons at the β position in **PP2** lie outside the macrocyclic ring current. However, the thienyl ring enhances resonance with the porphyrin core yielding a greater ring current. These β-protons therefore, will experience greater deshielding effect compared to those in **PP1** thus appear more downfield shifted. Further, the signals for protons on the thienyl substituent group lie at δ_H 7.66 and 7.49 (*d*, *J* = 3.6, 6.4 Hz) ppm integrating for a total of eight protons.

Elemental analysis results for **PP1** gave C 56.54, H 3.00, N 6.10. Analysis of the elemental results to determine the ratio of elements in the sample satisfied the assigned chemical formula $C_{44}H_{26}Br_4N_4$ of the target compound. MALDI-TOF MS spectra for **PP1** (Fig. S3) gave a molecular ion peak at *m/z* value of 930.94 against a calculated m/z value of 930.34 consistent with the molecular formula of the synthesized compound. Elemental analysis results for **PP2** found C

45.67, H 1.89, N 5.61, S 13.46 consistent with assigned chemical formula $C_{36}H_{16}Br_4S_4N_4$ of the target compound while the MS spectra (Fig. S4) provided an m/z value of 954.78 against a calculated m/z value of 954.42.

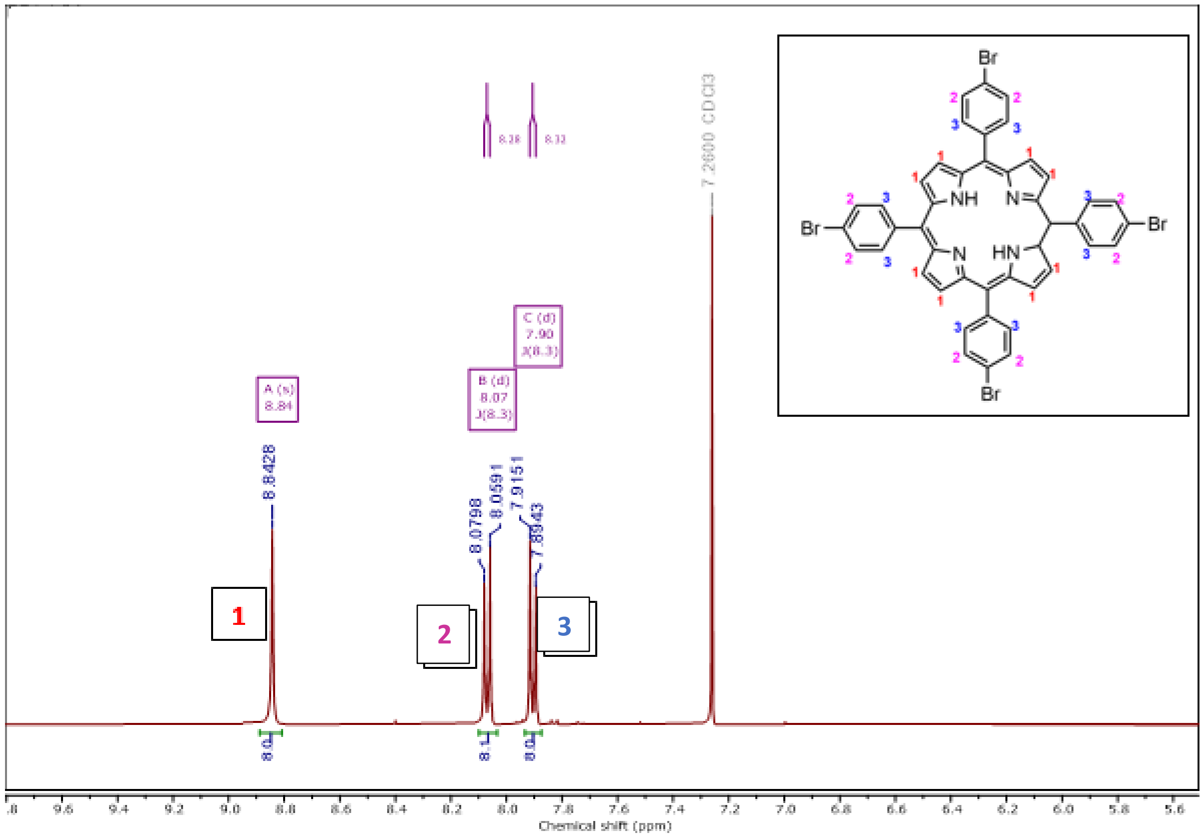

Figure S1: ^{1}H NMR spectrum for **PP1**.

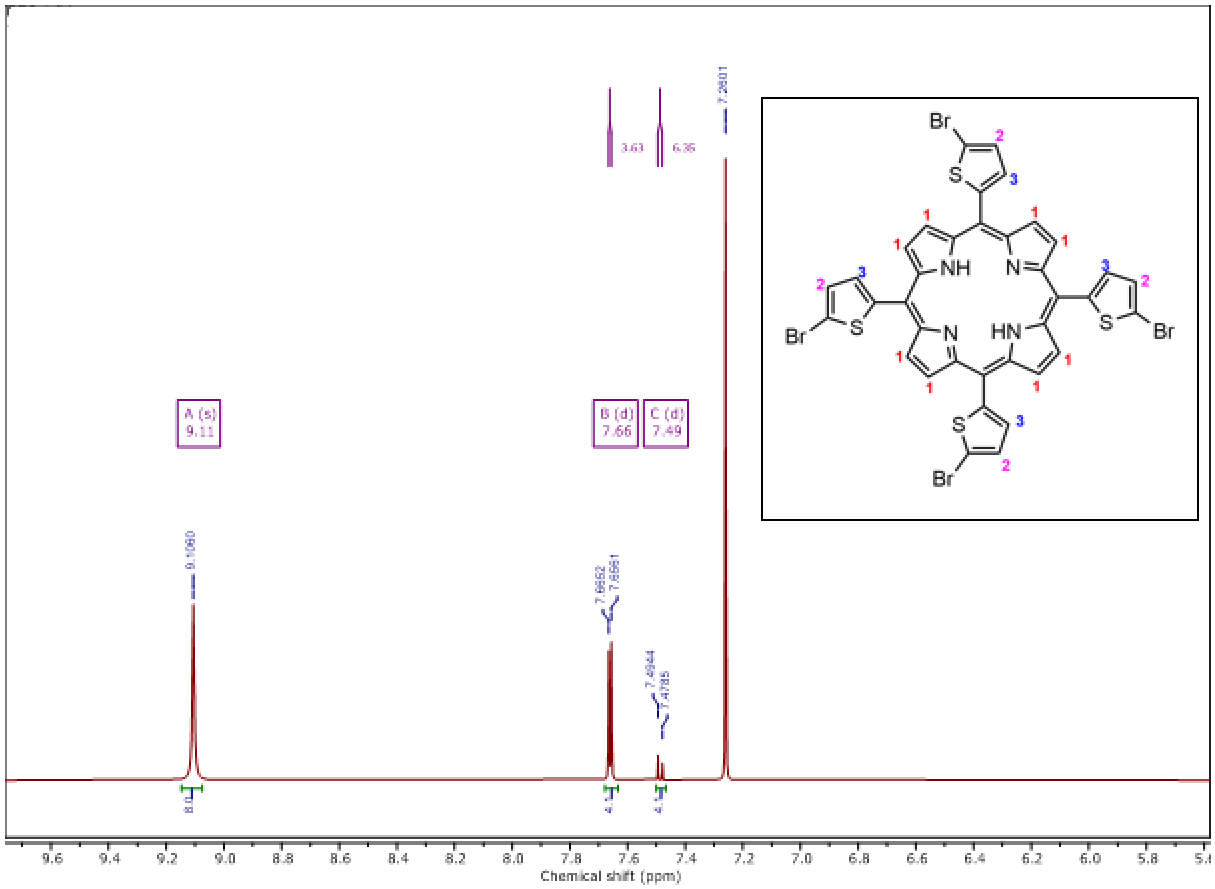

Figure S2: ^{1}H NMR spectrum for **PP2**.

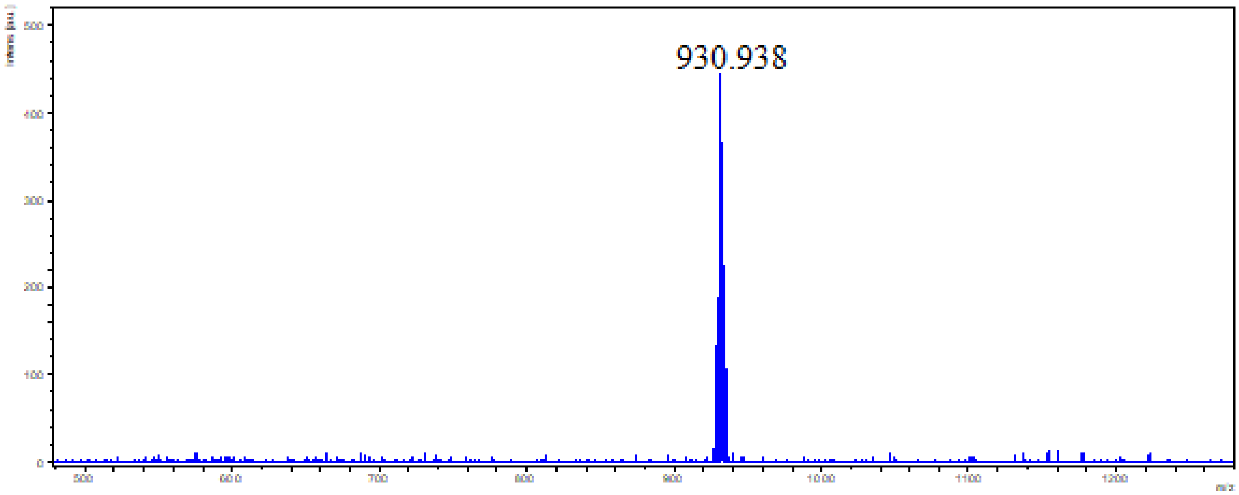

Figure S3: Mass spectrum for **PP1**.

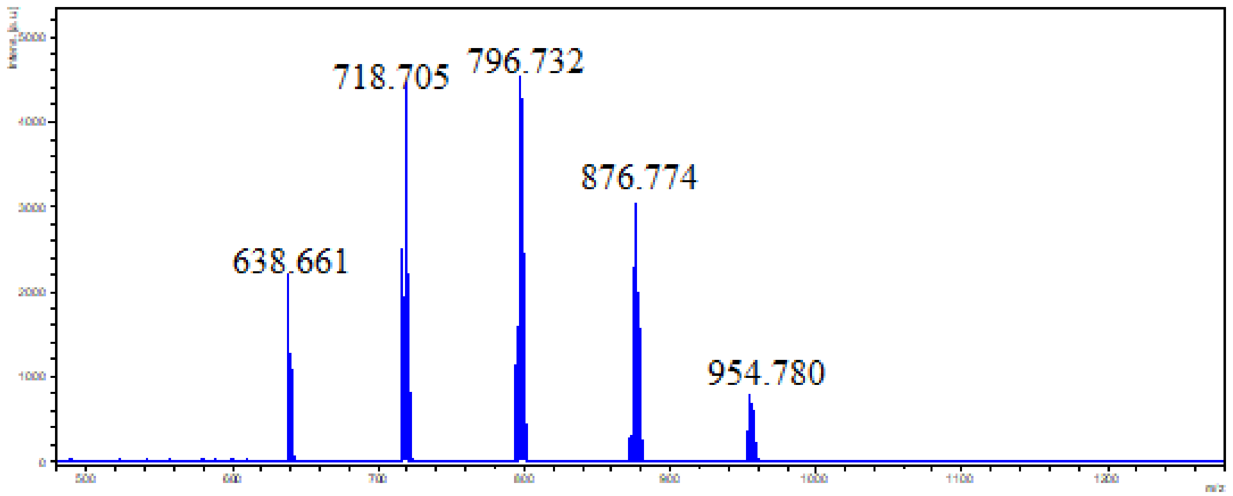

Figure S4: Mass spectrum for **PP2**.

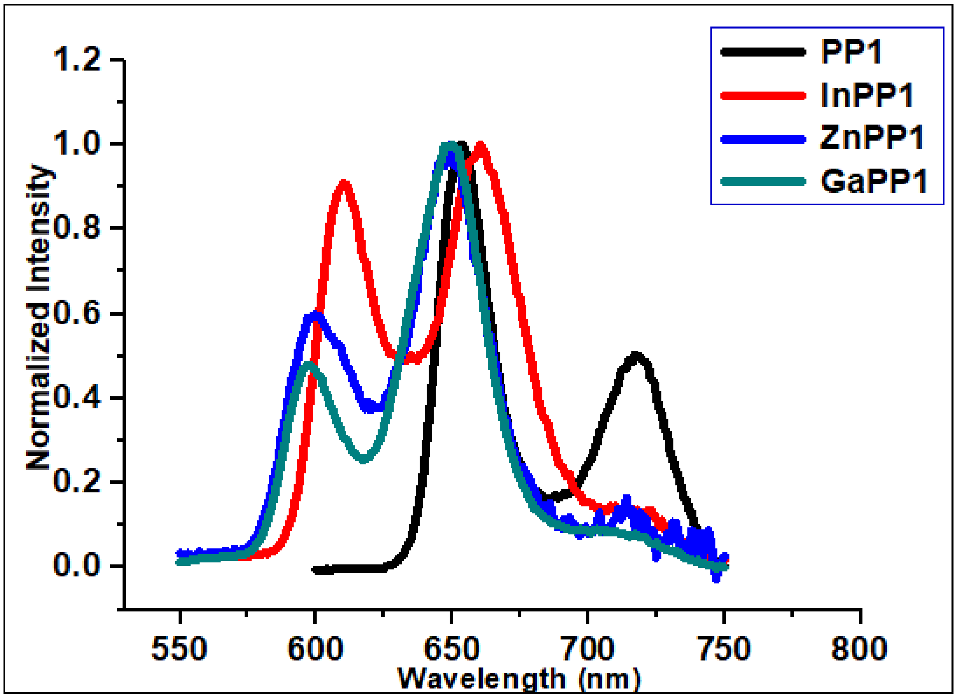

Figure S5: Fluorescence spectra of **PP1** and its metal complexes in toluene.

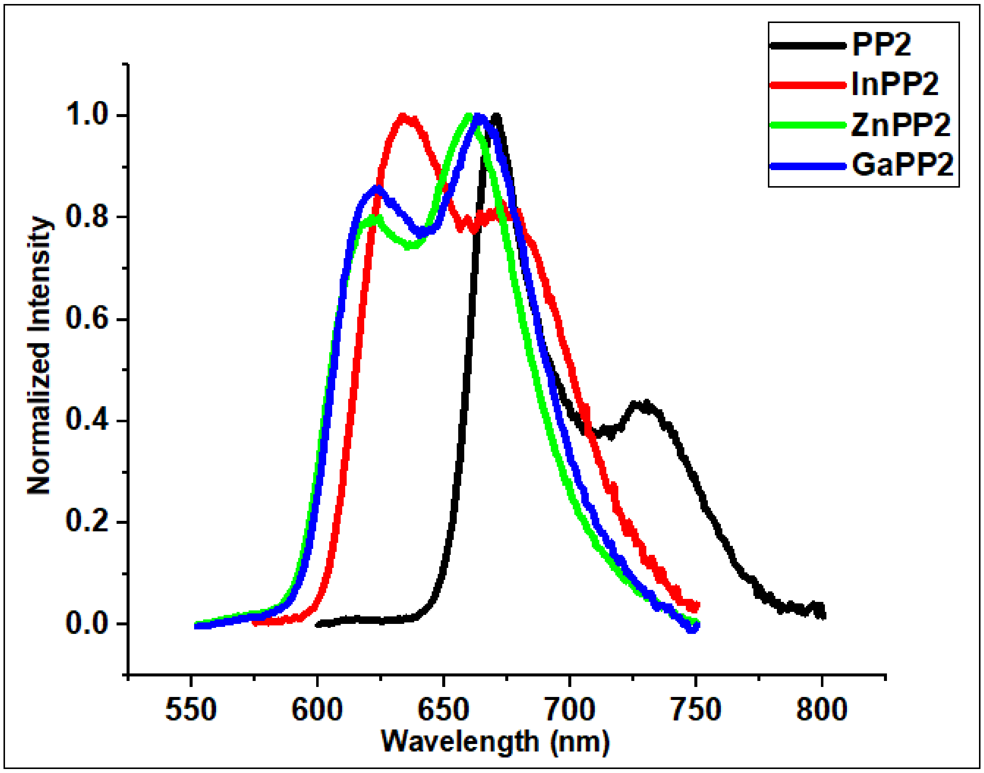

Figure S6: Fluorescence spectra of **PP2** and its metal complexes in toluene.

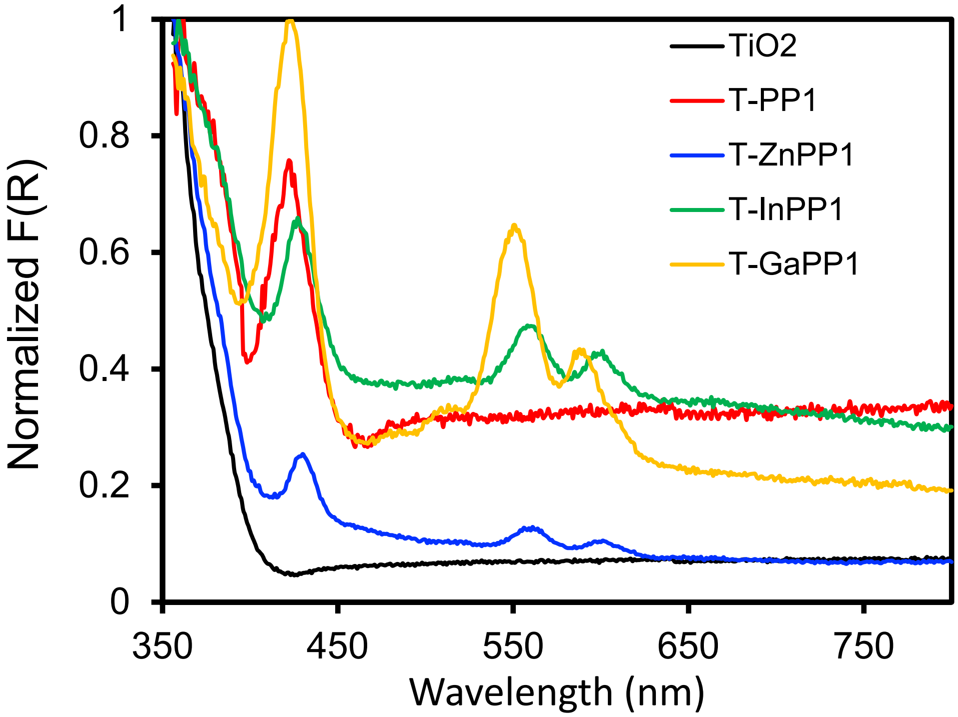

Figure S7: Normalized Diffuse Reflectance spectra for $\mathbf{TiO_2}$ on **PP1** and its metal complexes.

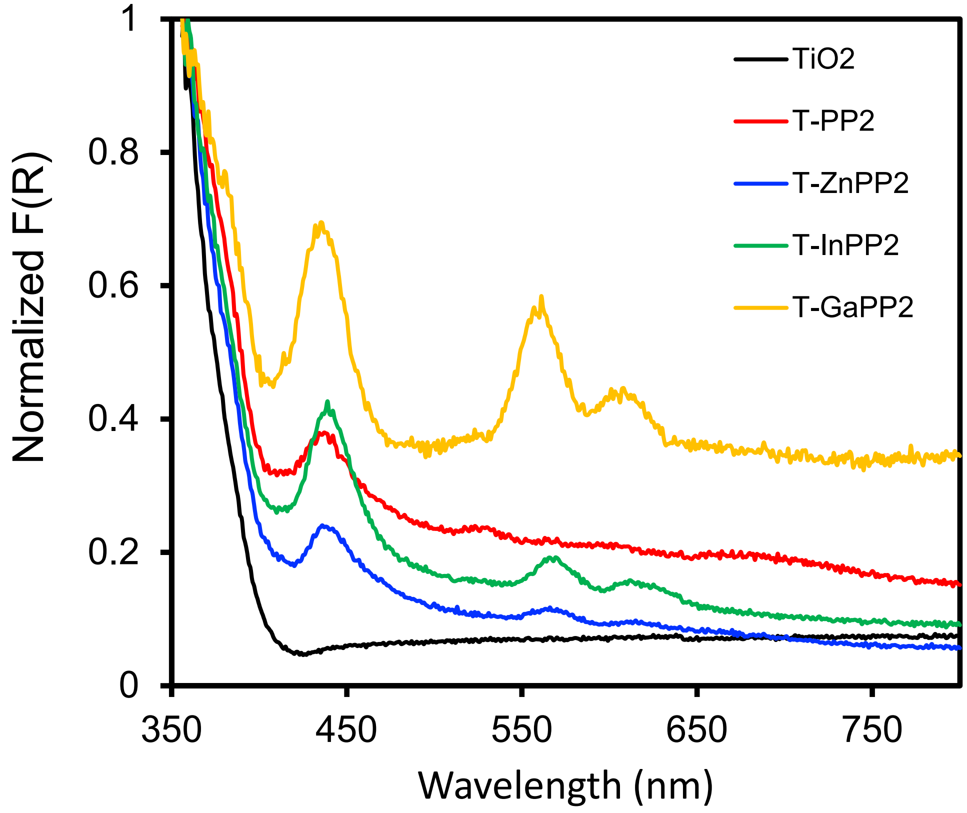

Figure S8: Normalized Diffuse Reflectance spectra for **TiO_2** on **PP2** and its metal complexes.
